# Crop rotations increased soil ecosystem multifunctionality by improving keystone taxa and soil properties in potatoes

**DOI:** 10.3389/fmicb.2023.1034761

**Published:** 2023-02-23

**Authors:** Qing-mei Li, Dai Zhang, Ji-zong Zhang, Zhi-jun Zhou, Yang Pan, Zhi-hui Yang, Jie-hua Zhu, Yu-hua Liu, Li-feng Zhang

**Affiliations:** ^1^Technological Innovation Center for Biological Control of Crop Diseases and Insect Pests of Hebei Province, Baoding, China; ^2^College of Plant Protection, Hebei Agricultural University, Baoding, China; ^3^College of Agronomy, Hebei Agricultural University, Baoding, China; ^4^Practice and Training Center, Hebei Agricultural University, Baoding, China

**Keywords:** rotation cropping, soil ecosystem multifunctionality, microbial community composition, keystone taxa, chemical properties, potato

## Abstract

Continuous cropping of the same crop leads to soil degradation and a decline in crop production, and these impacts could be mitigated through rotation cropping. Although crop rotation enhances soil fertility, microbial community diversity, and potato yield, its effects on the soil ecosystem multifunctionality (EMF) remain unclear. In the present research, we comparatively examined the effects of potato continuous cropping (PP) and rotation cropping [potato–oat rotation (PO) and potato–forage maize rotation (PFM)] on the soil EMF as well as the roles of keystone taxa, microbes abundance, and chemical properties in EMF improvement. It was demonstrated that soil EMF is increased in rotation cropping (PO and PFM) than PP. Soil pH was higher in rotation cropping (PO and PFM) than in PP, while total phosphorus (TP) and available phosphorus (AP) were significantly decreased than that in PP. Rotation cropping (PO and PFM) markedly changed the bacterial and fungal community compositions, and improved the potential plant-beneficial fungi, e.g., *Schizothecium* and *Chaetomium*, while reducing the abundances of the potentially phytopathogenic fungi, e.g., *Alternaria*, *Fusarium*, *Verticillium dahiae*, *Gibberella*, *Plectosphaerella*, *Colletotrichum*, *Phoma*, and *Lectera* in comparison with PP. Also, co-occurrence patterns for bacteria and fungi were impacted by crop rotation, and keystone taxa, e.g., *Nitrospira*.1, *Lysinibacillus*, *Microlunatus*.1, *Sphingomonas*.3, *Bryobacter*.1, *Micromonospora*, and *Schizothecium*, were enriched in PO and PFM than PP. The structural equation model (SEM) further demonstrated that cropping systems increased soil ecosystem multifunctionality through regulating SOM and keystone taxa (*Schizothecium*1), and keystone taxa were mediated by soil pH. This study suggested that rotation cropping might contribute to the improvement of soil ecosystem multifunctionality as well as the development of disease-suppressive soils in comparison with potato continuous cropping.

## 1. Introduction

Potato (*Solanum tuberosum*), one of the most important crops in the world, plays an irreplaceable role in ensuring world food security and promoting economic development (Gustavsen, 2021). Because of the limited cultivated area and economic interest, potato continuous cropping within the same field has become a very widespread problem (Zhou et al., 2019). Long-term continuous potato cropping results in soil-borne diseases, including common scab, black scurf disease, blackleg, and fusarium wilt, which lead to a reduction in potato productivity and sustainable health development (Xu et al., 2022), and biotic factors are the major causes for soil-borne diseases (Dias et al., 2015). In order to control these diseases, fungicides are extensively used in potato production to prevent and control those soil-borne diseases and maintain sufficient crop yield and product quality (Al-Mughrabi et al., 2015), while they can also cause serious risks to the environment and human health (Tan et al., 2020).

Rotation cropping is a safe and effective measure that can improve soil productivity, reduce pathogens, control plant soil-borne diseases, and increase yields in comparison with continuous cropping (Larkin and Halloran, 2014; Ashworth et al., 2020). Previous studies have suggested that potato–corn/green manure rotation provided higher tuber yield *via* enhancing abundances of some beneficial microbes (e.g., *Sphingomonas*, *Haliangium*, *Gemmatimonas*, and *Pseudogymnoascus*), while decreasing the abundances of pathogenic microbes (e.g., *Fusarium*, *Stagonosporopsis, Alternaria*, *Lectera, Fusaria*, and *Mortierell*) and autotoxic substances (Qin et al., 2017; Wang et al., 2022). Long-time rotation cropping improved microbial community composition and enhanced soil health which eventually contributed to improved plant growth. Thus, maintaining the potato production system is closely associated with improving diverse and functional soil microbial communities (Hiltunen et al., 2021).

Soil microbes (bacteria and fungi) play a crucial role in the agroecosystem, as they participate in material cycling and organic matter decomposition (Liu et al., 2019), and are vital and decisive factors in plant health and productivity (Guo et al., 2021). Keystone taxa play a role in biological connectivity and may be considered indicative markers of community migration and compositional rollover, which have the largest influence on microbial community and ecosystem functionality (Vick-Majors et al., 2014; Banerjee et al., 2016a,^2018^). Previous studies have demonstrated that keystone taxa can have significant effects on soil quality improvement, carbon transformation, and organic compound degradation (Banerjee et al., 2016b; Yan et al., 2019; Liu et al., 2022). Agricultural management, e.g., tillage practices, that effectively affect keystone taxa, also influence soil quality and ecosystem multifunctionality (Liu et al., 2022). However, the responses of soil ecosystem multifunctionality, microbial co-occurrence network, patterns, and keystone taxa to different cropping systems remain unclear.

Soil ecosystem multifunctionality motivated by soil microbes is important for maintaining the cycling of nutrients, the decomposition of organic matter, and plant productivity (Bardgett and van der Putten, 2014; Delgado-Baquerizo et al., 2016). Previous studies have shown that soil multifunctionality (e.g., C and N cycling) was affected by microbial community composition, diversity, and soil environment (e.g., pH and SOC) (Zheng et al., 2019). Agricultural management practices can enhance ecosystem services function and maintain ecosystem multifunctionality (Ryan et al., 2018). Recent studies have shown that intercropping can increase the soil ecosystem multifunctionality by improving available nutrients (Ma et al., 2022). It remains incompletely understood, however, whether the alterations in soil chemical properties affected by crop rotation affect the co-occurrence patterns of microbes and the relationship between keystone taxa and soil ecosystem multifunctionality.

In the present research, we comparatively explored the differences in soil chemical properties [pH, total nitrogen (TN), alkali hydrolyzable nitrogen (AN), organic matter (SOM), total phosphorus (TP), and available phosphorus (AP)], bacterial and fungal community compositions, co-occurrence patterns, keystone taxa, and ecosystem multifunctionality between potato continuous cropping (PP) and rotation cropping [potato–oat rotation (PO) and potato–forage maize rotation (PFM)]. The aims of the present study were to (1) investigate the responses of soil ecosystem multifunctionality, microbial community composition, co-occurrence network patterns, and keystone taxa to different cropping systems; (2) determine how the keystone taxa, microbes abundance, and chemical properties affect soil ecosystem multifunctionality under different cropping systems.

## 2. Materials and methods

### 2.1. Study site description

The study was conducted at the Zhangbei Agricultural Resource and Ecological Environment Key Field Research Station, Ministry of Agriculture and Rural Affairs, Zhangjiakou, Hebei, China (41°09’N, 114°42’E). The study site is situated at an elevation of 1,420 m, with a mean annual temperature of 3.9°C and a mean annual precipitation record of 382.5 mm. The soil type is meadow chestnut soil with a pH of 7.7, organic matter 18.53 g kg^–1^, alkaline hydrolysis nitrogen 80.68 mg kg^–1^, total nitrogen 1.09 g kg^–1^, available phosphorus 34.10 mg kg^–1^, total phosphorus 0.54 g kg^–1^, available potassium 76.63 mg kg^–1^, and total potassium 22.03 g kg^–1^ (Yao et al., 2020).

**FIGURE 1:**
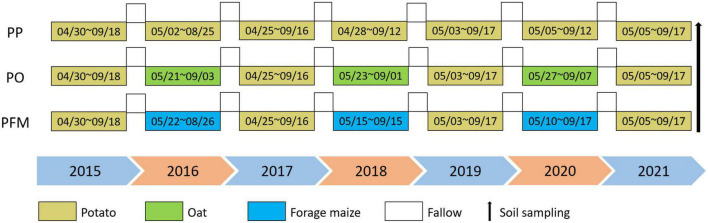
Schematic diagram of three experimental planting patterns.

Three treatments were used during the growing seasons from 2015 to 2021. Treatments used in this study include potato (*Solanum tuberosum*) continuous cropping (PP), potato–oat (*Avena sativa*) rotation (PO), and potato–forage maize (*Zea mays*) rotation (PFM). Three 20 m × 6 m experimental plots were established and treated as the three treatments described earlier. There were five pseudo-replicates within each experimental plot, and the size of each replicate plot was 4 m × 6 m. Fertilizers were used as basal fertilizers before sowing, with no irrigation throughout the crop growth period. The detailed experimental treatments are shown in Figure 1.

### 2.2. Soil sampling

After the harvest of potatoes, a total of 15 bulk soil samples (three treatments × five replicates) were collected with a 2-cm-diameter auger on 14 October 2021. For the bulk soils, 10 topsoil samples (0–20 cm) were randomly collected from each replicate plot and combined into a single sample (a replicate). Each composite sample (a replicate) was divided into two parts, where the first part was stored at –80°C before DNA extraction, and the second part was air-dried at room temperature for determining soil chemical properties [pH, total nitrogen (TN), alkali hydrolyzable nitrogen (AN), organic matter (SOM), total phosphorus (TP), and available phosphorus (AP)], enzyme activities [β-1,4-N-acetyl-glucosaminidase (NAG), β-1,4-glucosidase (β-GC), and alkaline phosphatase (ALP)].

### 2.3. Microbial DNA extraction, PCR amplification, and Illumina MiSeq

Total soil genomic DNA was extracted from 0.5 g of soil using the E.Z.N.A.^®^ soil DNA kit (Omega Bio-Tek, Norcross, GA, USA). The DNA extract was checked on 1% agarose gel, and DNA concentration and purity were determined with NanoDrop 2000 UV–vis spectrophotometer (Thermo Scientific, Wilmington, USA). Bacterial 16S rRNA gene fragments were performed using the general bacterial primers 338F–806R, which are specific to the V3–V4 hypervariable region (Wang et al., 2018). The ITS region was targeted with the primers ITS1F–ITS2R (Kerfahi et al., 2016). The adaptor and primer sequences were trimmed using the cutadapt plugin. The quality control and identification of amplicon sequence variants were performed using the DADA2 plugin (Callahan et al., 2016) according to the standard protocols by Majorbio Bio-Pharm Technology Co., Ltd. (Shanghai, China). Through quality trimming (using Btrim to remove sequencing adaptors and low-quality regions), merging, and clustering (using the CD-HIT algorithm), samples were rarefied to a depth of 52,460 and 38,494 sequences per sample of bacterial and fungal communities, respectively, and clustered into 47,678 and 38,064 operational taxonomic units (OTUs) of bacterial and fungal communities, respectively, by a 97% similarity cutoff using UPARSE version 7.1 (Edgar, 2013). The taxonomy of each OTU representative sequence was analyzed by RDP Classifier version 2.2 (Wang et al., 2007) using a confidence threshold of 0.7. The raw data of bacterial and fungal sequences were deposited into the NCBI Sequence Read Archive (SRA) database under the following accession numbers: SRR22669278-22669292 (bacteria), and SRR22703913-22703927 (fungi).

### 2.4. Soil chemical properties, enzyme activities, and soil ecosystem multifunctionality analysis

Soil pH was determined in a mixture of water and soil suspension (2.5:1) with an electrode method. TN was done by measuring the residual ammonia by the Kjeldahl method. AN was determined by the alkaline diffusion method. TP was determined by Mo–Sb anti-spectrophotometric method. AP was extracted by the diacid method and determined by the molybdenum–antimony colorimetry. SOM was measured by the potassium dichromate external heating method. Soil analyses (pH, TN, AN, TP, AP, and SOM) procedures were conducted as detailed by Bao (2000).

Enzymatic activities of NAG and β-GC were determined by the colorimetric method, and ALP was measured using the disodium phenyl phosphate colorimetric method (Sinsabaugh et al., 2008).

Soil multifunctionality was assessed based on three soil functional attributes associated with the carbon (C), nitrogen (N), and phosphorus (P) cycles. NAG, TN, and AN for the N cycle; β-GC and organic matter for the C cycle; ALP, TP, and AP for the P cycle. Single soil functions were normalized with Z-score transformation and averaged to calculate the multifunctionality (Guo et al., 2021).

### 2.5. Statistical analyses

One-way ANOVA was employed to determine the effects of different cropping systems (PP, PO, and PFM) on the soil chemical properties, multifunctionality, and the abundance of the potential plant-beneficial and phytopathogenic microbes, and significant differences were analyzed by Duncan’s new multiple differences test at a *P-*value of < 0.05. Data were tested for normality and homogeneity of variance before conducting ANOVA and were log-transformed when needed. The Pearson correlation coefficient was used to determine the possible association among soil microbes, soil chemical properties, and ecosystem multifunctionality. SPSS 22.0 software was used for statistical analyses.

Principal coordinate analysis (PCoA) was calculated by the “vegan 3.3.1” package in R. Linear discriminant analysis (LDA) effect size (LEfSe) was conducted to illustrate the biomarkers in each treatment. Those with an LDA score of ≥ 2.5 for bacteria and ≥ 4.0 for fungi were considered to be important biomarkers in each treatment.

Co-occurrence network analysis of microbial communities at the genus level using high-throughput sequencing data and the relative abundance of a genus of > 0.1% was used in the analyses. A correlation matrix was analyzed using the “psych” package in the R environment and the co-occurrence network visualization was achieved *via* Gephi (version 0.9.2). Spearman correlations between genera were performed, and the correlations with a coefficient of more than 0.6 and a *P*-value of less than 0.05 were applied. Microbial community networks were built according to MENAP (Wu et al., 2021a).^1^ The topological roles of individual nodes in the network were decided by the threshold values of *Zi* and *Pi* (Ling et al., 2016; Han et al., 2022). Nodes were classified into four categories: peripherals (*Zi* < 2.5 and *Pi* < 0.62), connectors (*Zi* < 2.5 and *Pi* > 0.62), module hubs (*Zi* > 2.5 and *Pi* < 0.62), and network hubs (*Zi* > 2.5 and *Pi* > 0.62). The nodes assigned to the network connector, module hub, and hub were the generalists that may be paralleled to key organisms in the microbial community as predicted by the network theory (Han et al., 2022).

A structural equation model was performed to assess the direct and indirect effects of cropping systems, soil pH, SOM, keystone taxa (*Schizothecium*1 abundance), and potentially phytopathogenic microbes on the soil ecosystem multifunctionality (C and N cycling) using IBM SPSS AMOS 21. Before the SEM analysis, we integrated the relative abundances of potentially phytopathogenic fungi [*Alternaria* (Wang et al., 2022; Xu et al., 2022), *Fusarium* (Zhang et al., 2017), *Verticillium dahliae* (Zhao et al., 2021), *Gibberella* (Li et al., 2022), *Plectosphaerella* (Xu et al., 2014), *Colletotrichum* (Cuevas-Fernández et al., 2022), *Phoma* (Wunsch and Bergstrom, 2011), and *Lectera* (Cannon et al., 2012)] through the principal component analyses (PCA). The first principal component (PC1) was used in the subsequent SEM analysis to represent soil pathogenic microbe abundance. Sufficient model fits of the structural equation models by χ^2^/*df* (1 ≤ χ^2^/*df* ≤ 3 and 0.05 < *P* ≤ 1.00) and root mean square error of approximation (0 ≤ RMSEA ≤ 0.08) were used (Delgado-Baquerizo et al., 2016). The standardized total effect of each variable on the soil ecosystem multifunctionality was also determined for the structural equation model.

## 3. Results

### 3.1. Soil ecosystem multifunctionality and chemical properties

The response of the changes in soil ecosystem multifunctionality to different cropping systems is presented in Table 1. Soil multifunctionality of crop rotation (PO and PFM) soils was higher than those of PP soils (*P* = 0.069). Specifically, soil multifunctionality related to the C cycle (*P* < 0.001) and single soil functions β-GC (*P* < 0.001) were increased in the crop rotation (PO and PFM) soils than those of PP soils. Soil multifunctionality related to the N cycle (*P* = 0.002) and single soil functions NAG (*P* < 0.001) were increased in the crop rotation (PO and PFM) soils than those of PP soils. In contrast, single functions TP (*P* < 0.001) and AP (*P* < 0.001) relating to the soil P cycle were decreased in the crop rotation (PO and PFM) soils than the PP soils. Compared to PP, crop rotation increased soil pH (*P* < 0.001), and PFM also increased pH relative to PO. PO, but not PFM, also increased SOM (*P* = 0.002), TN (*P* = 0.055), and C/N (*P* = 0.029) than PP.

**TABLE 1:** The soil chemical properties, enzyme activities, and multifunctionality under different crop rotations.

Soil properties	PP	PO	PFM	*F*	*P*
EMF	-0.25 ± 0.08a	0.12 ± 0.12a	0.13 ± 0.12a	3.365	0.069
C cycle	-0.97 ± 0.11b	0.65 ± 0.18a	0.32 ± 0.09a	33.522	<0.001
β-GC (nmol g^–1^ h^–1^)	0.4867 ± 0.0005b	0.4915 ± 0.0009a	0.4937 ± 0.0006a	20.285	<0.001
SOM (g kg^–1^)	20.78 ± 0.54b	23.52 ± 0.21a	21.56 ± 0.31b	11.098	0.002
N cycle	-0.75 ± 0.17b	0.37 ± 0.17a	0.38 ± 0.17a	10.719	0.002
NAG (nmol g^–1^ h^–1^)	229.33 ± 14.09b	396.44 ± 8.19a	444.44 ± 23.45a	37.526	<0.001
TN (g kg^–1^)	1.22 ± 0.01b	1.27 ± 0.01a	1.26 ± 0.01ab	3.739	0.055
AN (mg kg^–1^)	109.13 ± 3.63a	111.37 ± 2.54a	110.89 ± 2.19a	0.136	0.874
P cycle	0.96 ± 0.12a	-0.65 ± 0.06a	-0.31 ± 0.21a	2.666	0.110
ALP (nmol g^–1^ h^–1^)	479.39 ± 11.89a	466.26 ± 4.91a	479.39 ± 3.98a	2.666	0.110
TP (g kg^–1^)	0.78 ± 0.02a	0.64 ± 0.02b	0.65 ± 0.01b	17.668	<0.001
AP (mg kg^–1^)	50.59 ± 1.38a	35.09 ± 1.96b	34.56 ± 2.09b	19.736	<0.001
pH	7.73 ± 0.02c	7.82 ± 0.01b	7.87 ± 0.00a	16.851	<0.001
C/N	9.85 ± 0.27b	10.73 ± 0.16a	9.93 ± 0.15b	4.802	0.029

SOM, soil organic matter; TN, total nitrogen; AN, alkali-hydrolysable nitrogen; TP, total phosphorus; AP, available phosphorus; C/N, organic carbon/total nitrogen; β-GC, β-1, 4-glucosidase; NAG, β-1, 4-N-acetyl-glucosaminidase; ALP, alkaline phosphatase; C cycle, carbon cycle; N cycle, nitrogen cycle; P cycle, phosphorus cycle; EMF, soil ecosystem multifunctionality; PP, potato continuous cropping; PO, potato–oat rotation; PFM, potato–forage maize rotation. Data represent the mean ± *SD* (*n* = 5). The different lowercase letters in the same rows indicate significant differences among treatments at the *P* < 0.05 level (one-way ANOVA).

### 3.2. Composition of microbial community

We sequenced the V3–V4 region of the 16S rRNA gene for 15 samples and obtained a total of 980,851 high-quality sequence reads that ranged from 52,460 to 80,674, with an average read length of 417 bp. The fungal ITS sequences totaled 754,215, and the number of sequences obtained from each sample ranged from 38,494 to 73,983, with an average read length of 237 bp (Supplementary Table 1).

**FIGURE 2:**
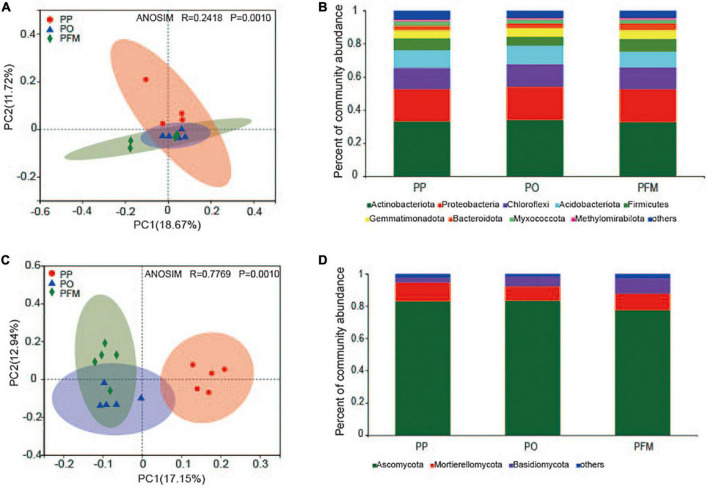
Principal coordinate analysis (PCoA) showing the changes in bacterial **(A)** and fungal **(C)** community composition. The abundances of total bacterial **(B)** and fungal **(D)** communities are based on the proportional frequencies of 16S rRNA and ITS sequences. PP, potato continuous cropping; PO, potato–oat rotation; PFM, potato–forage maize rotation.

The disparities in the structures of soil bacterial and fungal communities from different cropping systems were analyzed by PCoA, and the structures of the microbial communities among continuous cropping and rotational cropping were significantly different (Figure 2). The first two principal component axes explained 18.67% (PC1) and 11.72% (PC2) of the variation in the bacterial community. The PO and PFM were clustered together, and were separated from PP along the PC2 axis (ANOSIM *R* = 0.2418, *P* = 0.001) (Figure 2A). Similar to the soil bacterial community, crop rotation also changed the fungal community structure. The first two principal component axes explained 17.15% (PC1) and 12.94% (PC2) of the variation in the fungal community. The fungal communities in soil from the PP treatment were separated from that of the PO and PFM treatments along the PC1 axis, and the PO was separated from the PFM along the PC2 axis (ANOSIM *R* = 0.7769, *P* = 0.001) (Figure 2C).

**FIGURE 3:**
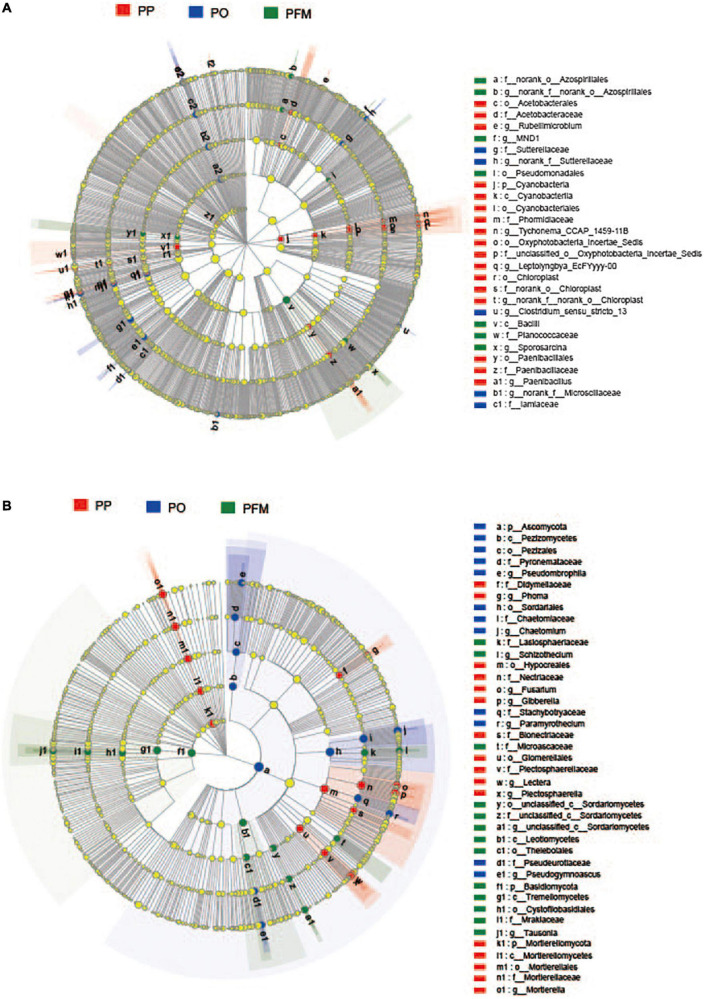
Cladogram showing the phylogenetic distribution of the bacterial **(A)** and fungal **(B)** lineages associated with soil from different crop rotations. Circles indicate phylogenetic levels from domain to genus. PP, potato continuous cropping; PO, potato–oat rotation; PFM, potato–forage maize rotation.

For the bacterial community, nine groups were described with an average relative abundance of > 1% at the phylum level. The dominant taxa in soil mainly included *Actinobacteriota* (33.32%), *Proteobacteria* (19.79%), *Chloroflexi* (13.20%), *Acidobacteriota* (10.40%), *Firmicutes* (6.79%), *Gemmatimonadota* (5.11%), *Bacteroidota* (3.20%), *Myxococcota* (2.37%), and *Methylomirabilota* (1.05%) (Figure 2B). Crop rotation shifted the dominant bacterial groups in comparison with PP. Dominant groups were displayed in cladograms, and LDA scores greater than or equal to 2.5 were confirmed by LEfSe (Figure 3A and Supplementary Figure 1A). *Cyanobacteria*, *Gemmatimonadetes*, and *Bacilli* performed major roles in PP, PO, and PFM, respectively. In addition, potential plant-beneficial bacteria *Bacillus* (*P* = 0.008) and *Pseudomonas* (*P* = 0.001), which are widely used for controlling plant diseases (Jiang et al., 2017; Wei et al., 2018), were in greater abundance in PFM than in PP and PO. In contrast, *Streptomyces scabiei*, which causes potato scab, was lower in abundance in PFM than in PP and PO, but there was no significant change between PP and PO (Table 2).

**TABLE 2:** Relative abundance (%) of beneficial and pathogenic microbes in different crop rotation systems.

Taxonomy	PP	PO	PFM	F	*P*
**Beneficial microbes**					
*Bacillus*	2.9011 ± 0.0989b	2.9087 ± 0.1194b	3.4885 ± 0.1419a	4.848	0.008
*Pseudomonas*	0.0306 ± 0.0019b	0.0306 ± 0.0023b	0.0461 ± 0.0024a	12.676	0.001
*Schizothecium*	0.7587 ± 0.0439c	0.9302 ± 0.1228b	1.4102 ± 0.1218a	12.924	0.001
*Chaetomium*	2.1264 ± 0.0779c	4.6924 ± 0.1191a	3.6452 ± 0.1547b	104.444	< 0.001
**Pathogenic microbes**					
*Streptomyces scabiei*	0.1493 ± 0.0105ab	0.1808 ± 0.0089a	0.1211 ± 0.0058b	7.840	0.008
*Verticillium dahliae*	0.1941 ± 0.0988a	0.0147 ± 0.0035b	0.0286 ± 0.0109b	7.204	0.009
*Alternaria*	1.2973 ± 0.0775a	0.6367 ± 0.1258b	0.8080 ± 0.0975b	9.000	0.004
*Fusarium*	3.1790 ± 0.6834a	1.0360 ± 0.0448b	1.0591 ± 0.0792b	7.647	0.007
*Gibberella*	4.7892 ± 0.7523a	1.9392 ± 0.1791b	1.5714 ± 0.2114b	11.583	0.002
*Plectosphaerella*	2.1767 ± 0.3430a	0.2462 ± 0.0310b	0.1654 ± 0.0188b	26.156	< 0.001
*Phoma*	0.9969 ± 0.1812a	0.0143 ± 0.0076b	0.0116 ± 0.0041b	23.548	< 0.001
*Lectera*	1.4917 ± 0.1317a	0.5599 ± 0.0549b	0.4197 ± 0.0574b	34.440	< 0.001
*Colletotrichum*	0.0576 ± 0.0144a	0.0096 ± 0.0043b	0.0238 ± 0.0059b	5.606	0.019

The relative species abundances were calculated as percentages of the total species abundances. Data represent the mean ± *SD* (*n* = 5). The different lowercase letters in the same rows indicate significant differences among treatments at the *P* < 0.05 level (one-way ANOVA). PP, potato continuous cropping; PO, potato–oat rotation; PFM, potato–forage maize rotation.

For the fungal community, the dominant phyla predominantly consisted of *Ascomycota* (81.36%), *Mortierellomycota* (10.30%), and *Basidiomycota* (6.19%) (Figure 2D). Crop rotation also shifted the dominant fungal groups in comparison with PP. Dominant groups were revealed in cladograms, and LDA scores greater than or equal to 4.0 were determined by LEfSe (Figure 3B and Supplementary Figure 1B). *Nectriaceae*, *Chaetomium*, and *Basidiomycota*, as the main dominant taxa, play key roles in PP, PO, and PFM, respectively. In addition, rotation cropping increased the potential plant-beneficial fungi abundance in comparison with PP (Table 2). Specifically, PO and PFM increased the abundance of *Schizothecium* (*P* = 0.001) and *Chaetomium* (*P* < 0.001) than PP, with PFM increasing *Schizothecium* to a greater degree, and PO increasing *Chaetomium* to a greater degree. Conversely, rotation cropping (PO and PFM) decreased the abundance of potentially phytopathogenic fungi than PP (Table 2). Specifically, the relative abundances of *Verticillium dahliae* (*P* = 0.009), *Alternaria* (*P* = 0.004), *Fusarium* (*P* = 0.007), *Gibberella* (*P* = 0.002), *Plectosphaerella* (*P* < 0.001), *Phoma* (*P* < 0.001), *Lectera* (*P* < 0.001), and *Colletotrichum* (*P* = 0.019) were significantly higher in PP than in PO and PFM but were insignificantly higher in PO and PFM.

### 3.3. Co-occurrence network patterns and keystone taxa analysis of soil microbial community

**FIGURE 4:**
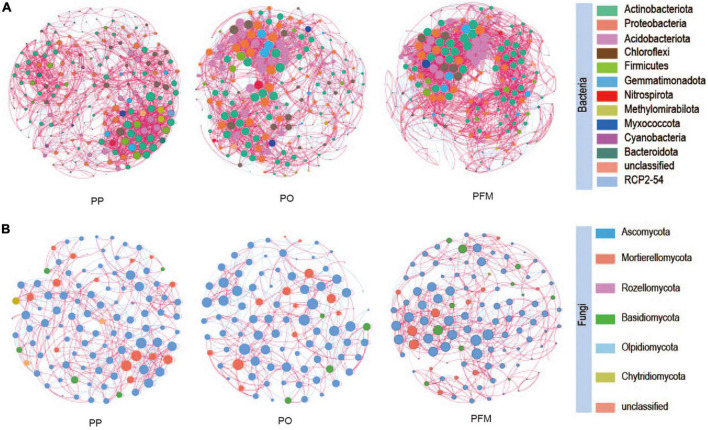
Co-occurrence network of soil bacteria **(A)** and fungi **(B)** in soil based on correlation analysis. A connection stands for a strong (Spearman’s *r* > 0.6) and significant (*P* < 0.05) correlation. The nodes represent a unique genus. The size of each node is proportional to the degree. The nodes are colored by taxonomy. PP, potato continuous cropping; PO, potato–oat rotation; PFM, potato–forage maize rotation.

In the present study, the interactions and differences of soil bacterial and fungal communities among different cropping systems were investigated at the genus level through co-occurrence networks (Figure 4), and the resulting complex pattern of the associations between nodes was depicted *via* calculating the topological properties (Supplementary Table 2). Significant differences in topological properties within bacterial and fungal networks were observed among the different cropping systems. For the bacterial community, the edges number, network density, modularity, and average clustering coefficient increased in PO and PFM than PP. The average path length decreased in PO and PFM than in PP. For the fungal community, compared with PP, the number of edges was increased in PFM and decreased in PO. The PO and PFM increased the network density while decreasing the modularity and average clustering coefficient than PP.

**FIGURE 5:**
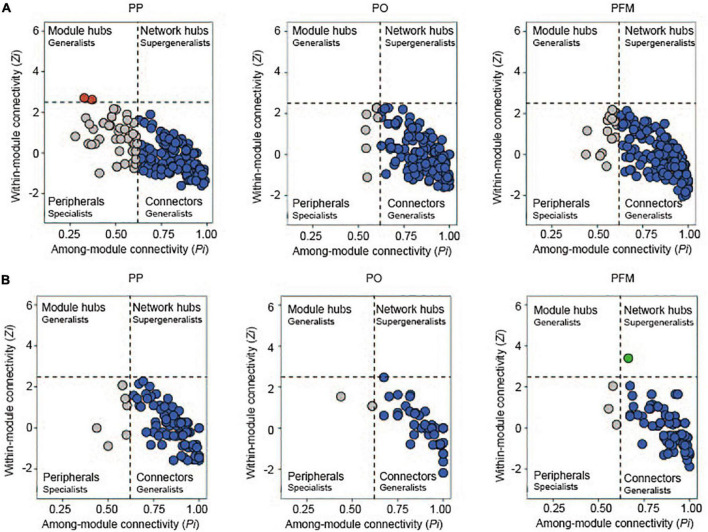
*Zi*–*Pi* plots of bacteria **(A)** and fungi **(B)** based on genus topological roles in bacterial and fungal networks. The threshold values of *Zi* and *Pi* for categorizing genus were 2.5 and 0.62, respectively. PP, potato continuous cropping; PO, potato–oat rotation; PFM, potato–forage maize rotation.

To determine keystone taxa in the networks, the connectivity of genera (nodes) was computed within (*Zi*) and among (*Pi*) modules. In this study, most genera were connectors with more links to the nodes within modules (Figure 5). Compared with PP, rotation cropping engaged more generalists, which connect different nodes within their modules. In the bacterial networks, 75.77% of genera for the PP network, 95.65% for the PO network, and 89.69% for the PFM network had connections with other nodes within and among modules (Supplementary Table 3). In the fungal networks, 93.58% of genera for the PP network, 95.16% for the PO network, and 95.24% for the PFM network had links to other nodes within and among modules (Supplementary Table 3). Crop rotation altered the keystone taxa in comparison with PP. Nodes, such as *Nitrospira*.1, *Lysinibacillus*, *Microlunatus*.1, *Sphingomonas*.3, *Bryobacter*.1 and *Micromonospora*, and *Schizothecium* were classified as network connectors (generalists) within crop rotation but peripherals (specialists) in PP (Figure 5 and Supplementary Table 4). In addition, one node (*Cystofilobasidium*) was classified as a network hub (supergeneralist) in the PFM network, whereas there were no nodes as supergeneralists in the PP network (Figure 5B and Supplementary Table 4).

Pearson’s correlation coefficient examined the relationships between the relative abundance of keystone taxa and soil properties (Supplementary Table 5). *Nitrospira*1 and *Sphingomonas*3 were significantly related to TN, while *Bryobacter*1 and *Cystofilobasidium* were closely correlated to C/N, and *Schizothecium*1 was significantly associated with pH, TN, TP, and AP.

### 3.4. Direct and indirect effects of soil biotic and abiotic factors on soil ecosystem multifunctionality

**FIGURE 6:**
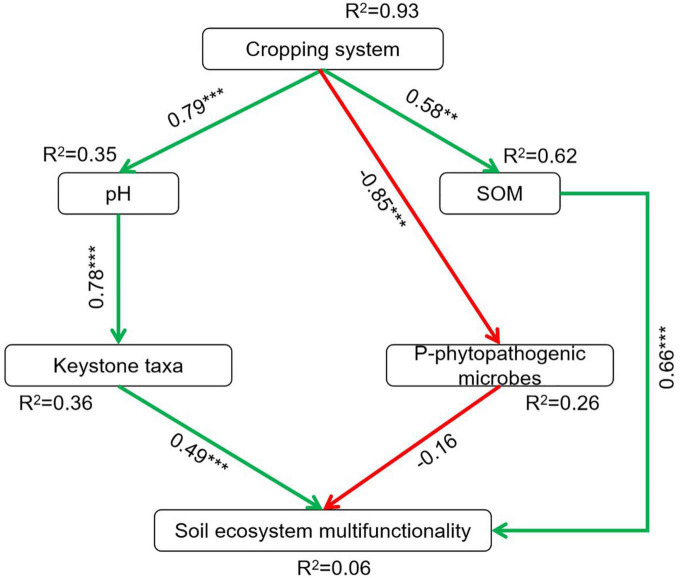
Structural equation modeling for the direct and indirect relationships of cropping systems on soil ecosystem multifunctionality. SOC, soil organic carbon. Red and green lines indicate significant negative and positive path coefficients, respectively; **P < 0.01, ***P < 0.001. χ^2^/df = 1.096, *P* = 0.362, RMSEA = 0.071. P-phytopathogenic microbes, potentially phytopathogenic microbes.

The relationships between soil biotic, abiotic factors, and soil ecosystem multifunctionality are shown in Supplementary Table 6. The soil ecosystem multifunctionality was strongly positively associated with pH (*r* = 0.64, *P* < 0.05), TN (*r* = 0.54, *P* < 0.05), AN (*r* = 0.62, *P* < 0.05), NAG (*r* = 0.66, *P* < 0.01), SOM (*r* = 0.68, *P* < 0.01), β-GC (*r* = 0.57, *P* < 0.05), *Chaetomiun* (*r* = 0.56, *P* < 0.05), *Schizothecium*1 (*r* = 0.53, *P* < 0.05), N cycle (*r* = 0.87, *P* < 0.01), and C cycle (*r* = 0.80, *P* < 0.01). The structural equation modeling (SEM) also estimated the association between cropping systems, soil chemical properties, microbes abundance (keystone taxa and potentially phytopathogenic microbes), and soil ecosystem multifunctionality (Figure 6 and Supplementary Table 7). The results indicated that the cropping systems had significant and direct positive effects on soil pH (*r* = 0.79, *P* < 0.001) and SOM (*r* = 0.58, *P* < 0.01), and significant and negative effects on potentially phytopathogenic microbes (*r* = –0.85, *P* < 0.001). However, the cropping systems had no direct effects on the keystone taxa and soil ecosystem multifunctionality. We observed that the pH (*r* = 0.78, *P* < 0.001) affected the keystone taxa abundance directly. Also, SOC (*r* = 0.66, *P* < 0.001) and keystone taxa abundance (*r* = 0.49, *P* < 0.001) were positively and closely linked with soil ecosystem multifunctionality, whereas the abundance of potentially phytopathogenic microbes was negatively associated with soil ecosystem multifunctionality (*r* = –0.16, *P* = 0.118).

## 4. Discussion

### 4.1. Effect of crop rotation on soil chemical properties and multifunctionality

The long-term continuous monocropping of crops such as potatoes, and soybean can cause severe soil degradation and nutrient imbalance (Liu et al., 2012; Wang et al., 2022). In the current research, as the years of potato continuous cropping increased, soil pH noticeably decreased due to the accumulation of phenolic acids that were beyond the processing capacity of soil microbes (Li et al., 2016; Liu et al., 2020). In addition, the composition of phenolic acids in root exudates or rhizosphere soils differed among different rotation crops (Zhou and Wu, 2018), this may be a possible reason leading to the pH difference between PO and PFM. SOM and TN remarkably increased in PO soil likely resulted due to larger root systems returning more residues to the soil in potato–oat rotation cropping system than in potato continuous cropping. Moreover, the nutrient uptake of different rotation crops and their utilization ability significantly differed. Plants with greater aboveground biomass often require more nutrients for growth than smaller plants. This may be a possible reason leading to the SOM and TN being higher in PO than that in PFM. Wu et al. (2021b) reported that the SOM and TN were higher in the wheat season (SOM 27.94∼30.31 g kg^–1^ and TN 0.92∼1.16 g kg^–1^) than that in the maize season (SOM 20.00∼24.26 g kg^–1^ and TN 0.89∼1.03 g kg^–1^). Conversely, the results of soil TP and AP were remarkably lower in crop rotation systems than that in continuous cropping of potatoes. The reason for this result may be due to different requirements of phosphorus among different crops and that the phosphorus requirement in maize and oats is greater than in potatoes.

### 4.2. Effect of crop rotation on soil microbial community composition

The microbial community structure was markedly affected by rotation cropping based on the PCoA. We found that the communities of bacteria and fungi were predominantly subdivided into two groups, and PP varied from those of PO and PFM. The result indicates that rotation cropping is a major reason for determining the changes in bacterial and fungal community composition (Liu et al., 2020). This is possibly attributed to crop root exudates and residual accumulation differences in a soil environment with different cropping systems because crop root exudates and residues can impact the structure of microbial communities by providing different nutritional substances for microbes. In addition, communities in the soil of the PO and PFM clustered together for bacteria but were separated by the PC2 axis for fungi. This result indicated that different rotation crops affected the fungal community structures. Rotation cropping changed the dominant microbes in comparison with potato continuous cropping. LEfSe analysis suggested that the variation in the bacterial community structure was mainly driven by 26 taxa, among which *Cyanobacteria*, *Gemmatimonadetes*, and *Bacilli* performed a major role in PP, PO, and PFM, respectively. *Gemmatimonadetes* are copiotrophic populations and prefer decomposing labile organic carbon fractions with rich nutrients (Ghosh et al., 2016; Clocchiatti et al., 2020) and then obtained higher abundance in PO soil with greater TN and SOM. In this study, the abundance of *Gemmatimonadetes* showed a significant and positive association with TN (Supplementary Table 5). The C/N increased in the soil after the maize straw returned. The *Bacillaceae* family that belongs to the class *Bacilli* had strong resistance to harmful external factors, and the *Bacillus* genus can effectively decompose organic matter, playing an important role in the element cycle in ecosystems (Wu et al., 2021a). In this study, the abundance of *Bacilli* showed a significant and positive association with C/N (Supplementary Table 5). In addition, the abundance of *Bacillus* and *Pseudomonas* increased in PFM than PP and PO, and some species within *Bacillus* and *Pseudomonas* are antagonistic strains to plant pathogens, allowing potato and banana to suppress common scab and fusarium wilt disease (Lin et al., 2018; Tao et al., 2020). Li et al. (2022) documented that, compared with peanut continuous cropping, rape–peanut–winter wheat–summer maize rotation increased *Bacillus* abundance, which has biological control activities. However, the pathogenic bacteria *Streptomyces scabiei*, which causes potato scab diseases, was slightly decreased in PFM than PP and PO. This result may be associated with the increased abundance of potential plant-beneficial bacteria, which inhibit the growth and sporulation of *Streptomyces scabiei* within PFM. Previous studies reported that both *Pseudomonas fluorescens* and *Bacillus amyloliquefaciens* can inhibit the growth of *Streptomyces scabiei* and reduce the occurrence of potato common scab in potato production (Arseneault et al., 2015; Lin et al., 2018).

With regards to fungi, *Nectriaceae*, *Chaetomium*, and *Basidiomycota*, as the main dominant taxa, performed major roles in PP, PO, and PFM, respectively. *Nectriaceae* contain pathogens that cause the rotting of plant roots (Toju et al., 2018). Species of *Chaetomium* are important agents of cellulose degradation (Wu et al., 2021a); the degradation of oat residues may lead to a higher abundance of *Chaetomium*. *Basidiomycota* contains many saprotrophic soil fungi that are involved in aerobic cellulose degradation (Boer et al., 2005), which may improve soil fertility. In addition, the abundance of *Schizothecium* was significantly higher in PFM than that in PP and PO, *Chaetomium* was significantly higher in PO than that in PP and PFM, and these genera can protect crops and vegetables from diseases (Zhao et al., 2013; Nong et al., 2017). There were significant differences in soil environment among different cropping ecosystems. This could be a reason to explain the fungal community composition differences among different cropping ecosystems. Soil pH and nutrients are important factors affecting fungal community composition (Wang et al., 2022). In this study, the abundance of *Chaetomium*, *Schizothecium*, and *Basidiomycota* showed significant and positive associations with pH and TN, respectively, while significant negative correlations between the abundance of *Nectriaceae* and pH and TN were observed (Supplementary Table 5). Other studies demonstrated that the difference in the fungal community composition among different treatments is also caused by the root exudates (e.g., sugars, organic acids, aromatics, and enzymes) of the rotation crops (Lang et al., 2019). Conversely, the abundance of potentially phytopathogenic fungi was significantly enriched in PP. *Alternaria* is a potentially phytopathogenic fungus that causes potato early blight disease, potato brown spot, and soybean black spot, and was significantly richer in PP compared with that in PO and PFM (Wang et al., 2022; Xu et al., 2022). *Fusarium* can infect a wide variety of crops and lead to corresponding diseases, such as potato dry rot disease and potato fusarium wilt (Zhang et al., 2017), which was higher in PP than that in PO and PFM. *Verticillium dahliae* can cause verticillium wilt in the potato during growth (Zhao et al., 2021) and was enriched in PP than PO and PFM.

These results suggested that continuous potato cropping promotes the growth and proliferation of specific pathogenic microbes in the soil, enhances disease infection risk, and disturbs the balance of the microbial community structure, while the rotation cropping of potato and oat (PO) and potato and forage maize (PFM) reduced potentially phytopathogenic microbes but increased potential plant-beneficial microbes. This may be the major reason that rotation cropping increased the yield and decreased the diseases of potatoes than continuous cropping of potatoes (Yao et al., 2020). Variations of soil microbial community composition in response to different cropping systems also revealed that rotation cropping in comparison with continuous cropping of potatoes can maintain soil ecosystem health.

### 4.3. Effect of crop rotation on co-occurrence network patterns and keystone taxa

Core microbes may represent coevolution with plants, which may be significant for plant health and productivity (Cúcio et al., 2016). In the current study, we further explored the interactions and differences in both bacterial and fungal co-occurrence patterns under different cropping systems by analyzing co-occurrence networks. Network topological properties displayed obvious distinctions in both bacterial and fungal co-occurrence patterns among PP, PO, and PFM. The number of edges for bacterial communities in rotation cropping soils was dramatically greater than those in potato continuous cropping soils. This finding is in line with Liu et al. (2020), who reported that the number of edges increased in the network of maize–soybean rotation than soybean continuous cropping. This result indicated that rotation cropping exhibits a larger network size and recruits more microbes participating in the bacteria–bacteria interactions than those in potato continuous cropping (Karimi et al., 2019). The PO and PFM microbial networks had larger network densities and lower average path lengths than the network of PP. This indicated that, compared with potato continuous cropping, rotation cropping enhanced the bacteria–bacteria interactions in terms of exchanges of nutrients, information, and energy among different communities (Chen et al., 2018; Yan et al., 2021). In addition, the average clustering coefficient and modularity were greater in PO and PFM networks than that in the PP network, indicating that bacteria in rotation cropping soils are more sensitive to the disturbance of external environmental factors and respond more rapidly, and community structure is more prone to change (de Araujo et al., 2019). These findings are in line with Chen et al. (2018), who reported that there were higher connectivity and clustering coefficient in the tobacco–corn rotation network than in tobacco continuous cropping. For the fungal community, changes in the number of edges and network density as well as average path length were consistent with alterations in the bacterial communities. However, the average clustering coefficient and modularity showed contrary changes in the bacterial community. This may be primarily attributed to the slow responses of soil fungi to external environmental change and may have less influence on the whole ecological network of fungi within brief periods (Wu et al., 2021a).

Rotation cropping altered the keystone taxa in both bacterial and fungal co-occurrence networks, which play important roles in the structure of the microbial community. In networks, generalists are beneficial for sustaining the microbial community balance and are emerging as essential players in enhancing the exchanges of information, materials, and energy among species in networks (Chen et al., 2018; Yan et al., 2021). For bacterial networks, the *Zi*–*Pi* relationship of every individual genus demonstrated that 95.65 and 89.69% of generalists existed in the networks of PO and PFM, respectively. However, in the PP network, the relative abundance of generalists decreased to 75.77% (74.74% connectors and 1.03% module hubs). For fungal networks, generalists that existed in the networks of PO and PFM were 95.16 and 95.24%, respectively. In addition, 1.19% of supergeneralists existed in the PFM network, whereas the relative abundance of generalists decreased to 93.58% in the PP network. Chen et al. (2018) reported that there were more generalists in the tobacco–corn rotation network than in tobacco continuous cropping. Yang et al. (2021) found that the number of connectors increased under pulse frequency (e.g., pea–pea–pea–wheat) than low-pulse frequency (e.g., pea–wheat–wheat–wheat) in crop rotations. The higher number of generalists under rotation cropping networks indicated that soil microbes were more active within their own modules under continuous cropping, but tended to establish connections with genera located at different modules under crop rotation. These results revealed that rotation cropping enhanced the interactions of soil microbes and altered the roles of some nodes and modified the ecological functions of key microbes in soils in comparison with potato continuous cropping. Thus, the reduction in the number of generalists and supergeneralists after potato continuous cropping could be perceived as a major reason causing the problems associated with continuous cropping in potatoes.

Crop rotation altered the distribution of keystone taxa (Supplementary Table 4). Specifically, *Nitrospira*.1 was the node with a maximum degree and was determined as a generalist in the PO and PFM networks, but was determined as a specialist in the PP network. *Nitrospira* plays an important role in nitrogen cycling (Daims et al., 2015; Ochieno et al., 2021). In this study, *Nitrospira*.1 was positively associated with TN (Supplementary Table 5). Some nodes that were identified as generalists [*Lysinibacillus* (*Firmicutes*), *Microlunatus*.1 (*Actinobacteriota*), and *Sphingomonas*.3 (*Proteobacteria*)] in the PO and PFM networks but as specialists in the PP network play key roles in the degradation of pesticide and organic pollutants (Li et al., 2007, 2018). Species of *Firmicutes* and *Actinobacteriota* could produce antibacterial and nematocidal compounds to prevent some soil-borne diseases, such as soybean root rot and potato scab (Qi et al., 2010; Sugiyama et al., 2014; Shi et al., 2019). Phyla *Proteobacteria* is a copiotrophic population and prefer decomposing labile organic C fractions with rich nutrients (Ghosh et al., 2016; Clocchiatti et al., 2020). Previous studies demonstrated that the phylum *Actinobacteriota* was the most important keystone member in potato soils (Gu et al., 2022). Chen et al. (2018) reported that microbes belonging to *Firmicutes* and *Proteobacteria* were enriched in the tobacco–corn rotation network. Oberholster et al. (2018) found that members of the *Proteobacteria* are the most prominent keystone taxa in the sunflower–sorghum rotation networks. In this study, *Sphingomonas*.3 belonging to *Proteobacteria* was positively associated with TN (Supplementary Table 5). *Bryobacter*.1 was the node with a maximum degree and was classified as a generalist in the PO network, but was classified as a specialist in the PP network. Liu et al. (2020) reported that *Bryobacter aggregates*, which have the ability to decompose organic matter in the soil, were classified as keystone taxa in maize–soybean rotation. In this study, *Bryobacter*.1 was positively associated with C/N (Supplementary Table 5). *Micromonospora* (*Actinobacteriota*) and *Schizothecium* (*Ascomycota*) were the nodes with maximum degrees and were classified as generalists in the PFM network, but were classified as specialists in the PP network. In addition, one node [*Cystofilobasidium* (*Basidiomycota*)] was classified as a network hub (supergeneralist) in the PFM network, whereas was not classified as a supergeneralist in the PP network. All those genera are considered beneficial microbes and have been used to control crop diseases in agricultural production (Hirsch and Valdés, 2010; Garat et al., 2010; Liu et al., 2020). This result is contrary to a previous study, which reported that *Actinobacteriota*, *Basidiomycota*, and *Ascomycota* were the most important keystone taxa in soils with potatoes cropping (Hou et al., 2020). This may be related to the difference in soil environment and nutrient resources. Correlation analysis indicated that significant positive associations occur among *Schizothecium*1, pH, and TN (Supplementary Table 5), and negative correlations among *Schizothecium*1, TP, AP (Supplementary Table 5), *Alternaria*, and other potentially phytopathogenic fungi (Supplementary Table 8). In addition, keystone taxa *Cystofilobasidium* exhibited a significantly negative correlation with *Streptomyces scabiei* (Supplementary Table 8). Therefore, these keystone functional taxa in PO and PFM may be beneficial for improving soil ecosystem environments and enhancing soil disease-suppression ability after long-term rotation cropping in comparison with continuous cropping of potatoes.

### 4.4. Factors mediating soil ecosystem multifunctionality under cropping systems

Soil chemical properties not only affected microbial community compositions but also the soil ecosystem multifunctionality. In this study, soil ecosystem multifunctionality was increased in crop rotation than PP, mainly driven by pH, TN, AN, SOM, N cycle, and C cycle. This indicates that higher carbon and nitrogen resource availability favors the growth of microbes, eventually promotes biogeochemical cycles, and enhances ecological functions (Geyer et al., 2016; Han et al., 2021). Also, soil biotic factors affect soil ecosystem multifunctionality. A previous study has demonstrated that microbes play critical roles in supporting ecosystem functioning (Delgado-Baquerizo et al., 2016). In this study, positive links among keystone taxa, potential plant-beneficial microbes, and soil ecosystem multifunctionality are conspicuous. *Schizothecium*1 and *Chaetomium* were positively related to C and N cycling as well as soil ecosystem multifunctionality (Supplementary Table 6), and the potential phytopathogenic microbes were negatively associated with C and N cycling as well as soil ecosystem multifunctionality (Supplementary Table 6). SEM also suggested that pH and SOC were abiotic factors affecting soil ecosystem multifunctionality (Figure 6), and that pH indirectly affected soil ecosystem multifunctionality by mediating keystone taxa, and SOC directly affected soil ecosystem multifunctionality. Soil pH decreased with the increasing years of continuous cropping (Li et al., 2016), which is an important factor significantly influencing soil microbial community composition (Wang et al., 2019), and subsequently affecting soil ecosystem multifunctionality. Rotation crop residues are a source of organic carbon and can increase organic carbon input (Rao et al., 2021), and then promote soil biochemistry processes and ecosystem multifunctionality (Ma et al., 2022).

## 5. Conclusion

Our experimental findings demonstrated that rotation cropping (PO and PFM) altered soil ecosystem multifunctionality, chemical properties, microbial community compositions, and keystone taxa in comparison with potato continuous cropping. In addition, compared with potato continuous cropping, rotation cropping increased the abundance of potential plant-beneficial bacteria and fungi but reduced potentially phytopathogenic bacteria and fungi, indicating that rotation cropping causes a more healthy microflora, and is beneficial to soil health and sustainable use of soil. Furthermore, co-occurrence networks of bacteria within rotation cropping (PO and PFM) and co-occurrence networks of fungi within PFM were more complex than potato continuous cropping. Keystone taxa were related to bacterial and fungal functional groups that may play underlying roles in the nutrient cycling, toxic material degradation, and prevention and control of soil-borne disease, suggesting that these keystone taxa may play vital roles in improving the soil environment and ecosystem multifunctionality and may make it possible to develop disease-suppressive soils in rotation cropping systems. Collectively, rotation cropping is an effective practice to improve soil ecosystem multifunctionality in agroecosystems and relieve continuous cropping obstacles in comparison with potato continuous cropping, and this study provides a scientific basis for the selection of rotation crops in potato continuous cropping.

## Data availability statement

The original contributions presented in this study are included in the article/Supplementary material, further inquiries can be directed to the corresponding authors.

## Author contributions

J-HZ: conceptualization, funding acquisition, project administration, supervision, and validation. Q-ML: data curation, formal analysis, software, and writing—original draft. Q-ML, J-ZZ, Y-HL, and L-FZ: investigation. J-HZ, Q-ML, and Z-JZ: methodology. DZ, YP, and Z-HY: resources. Q-ML, J-HZ, and J-ZZ: writing—review and editing. All authors contributed to the article and approved the submitted version.

## Footnotes

1
http://ieg2.ou.edu/MENA/main.cgi

## References

[B1] Al-Mughrabi K. I. Vikram A. Poirier R. Jayasuriya K. Moreau G. (2015). Management of common scab of potato in the field using biopesticides, fungicides, soil additives, or soil fumigants. *Biocontrol Science and Technology* 26 125–135. 10.1080/09583157.2015.1079809

[B2] Arseneault T. Goyer C. Filion M. (2015). *Pseudomonas* fluorescens LBUM223 increases potato yield and reduces common scab symptoms in the field. *Phytopathology* 105 1311–1317. 10.1094/PHYTO-12-14-0358-R 25961336

[B3] Ashworth A. Owens P. Allen F. (2020). Long-term cropping systems management influences soil strength and nutrient cycling. *Geoderma* 361 114062. 10.1016/j.geoderma.2019.114062

[B4] Banerjee S. Baah-Acheamfour M. Carlyle C. N. Bissett A. Richardson A. E. Siddique T. (2016a). Determinants of bacterial communities in C anadian agroforestry systems. *Environmental Microbiology* 18 1805–1816. 10.1111/1462-2920.12986 26184386

[B5] Banerjee S. Kirkby C. A. Schmutter D. Bissett A. Kirkegaard J. A. Richardson A. E. (2016b). Network analysis reveals functional redundancy and keystone taxa amongst bacterial and fungal communities during organic matter decomposition in an arable soil. *Soil Biology and Biochemistry* 97 188–198. 10.1016/j.soilbio.2016.03.017

[B6] Banerjee S. Schlaeppi K. van der Heijden M. G. (2018). Keystone taxa as drivers of microbiome structure and functioning. *Nat Rev Microbiol* 16 567–576. 10.1038/s41579-018-0024-1 29789680

[B7] Bao S. (2000). *Soil agro-chemistries analysis.* Beijing: Agricultural Press.

[B8] Bardgett R. D. van der Putten W. H. (2014). Belowground biodiversity and ecosystem functioning. *Nature* 515 505–511. 10.1038/nature13855 25428498

[B9] Boer W. D. Folman L. B. Summerbell R. C. Boddy L. (2005). Living in a fungal world: impact of fungi on soil bacterial niche development. *FEMS microbiology reviews* 29 795–811. 10.1016/j.femsre.2004.11.005 16102603

[B10] Callahan B. J. McMurdie P. J. Rosen M. J. Han A. W. Johnson A. J. A. Holmes S. P. (2016). DADA2: High-resolution sample inference from Illumina amplicon data. *Nat. Methods* 13 581–583. 10.1038/nmeth.3869 27214047 PMC4927377

[B11] Cannon P. Buddie A. Bridge P. de Neergaard E. Lübeck M. Askar M. (2012). Lectera, a new genus of the Plectosphaerellaceae for the legume pathogen Volutella colletotrichoides. *MycoKeys* 3 23–36. 10.3897/mycokeys.3.3065

[B12] Chen S. Qi G. Luo T. Zhang H. Jiang Q. Wang R. (2018). Continuous-cropping tobacco caused variance of chemical properties and structure of bacterial network in soils. *Land Degrad Dev* 29 4106–4120. 10.1002/ldr.3167

[B13] Clocchiatti A. Hannula S. E. van den Berg M. Korthals G. De Boer W. (2020). The hidden potential of saprotrophic fungi in arable soil: Patterns of short-term stimulation by organic amendments. *Applied Soil Ecology* 147 103434. 10.1016/j.apsoil.2019.103434

[B14] Cúcio C. Engelen A. H. Costa R. Muyzer G. (2016). Rhizosphere microbiomes of European seagrasses are selected by the plant, but are not species specific. *Front Microbiol* 7:440. 10.3389/fmicb.2016.00440 27065991 PMC4815253

[B15] Cuevas-Fernández F. B. Robledo-Briones A. M. Baroncelli R. Trkulja V. Thon M. R. Buhinicek I. (2022). First report of Colletotrichum graminicola causing maize anthracnose in Bosnia and Herzegovina. *Plant Disease* 103 3281. 10.1094/PDIS-06-19-1224-PDN

[B16] Daims H. Lebedeva E. V. Pjevac P. Han P. Herbold C. Albertsen M. (2015). Complete nitrification by Nitrospira bacteria. *Nature* 528 504–509. 10.1038/nature16461 26610024 PMC5152751

[B17] de Araujo A. S. F. Miranda A. R. L. Sousa R. S. Mendes L. W. Antunes J. E. L. de Souza Oliveira L. M. (2019). Bacterial community associated with rhizosphere of maize and cowpea in a subsequent cultivation. *Appl Soil Ecol* 143 26–34. 10.1016/j.apsoil.2019.05.019

[B18] Delgado-Baquerizo M. Maestre F. T. Reich P. B. Jeffries T. C. Gaitan J. J. Encinar D. (2016). Microbial diversity drives multifunctionality in terrestrial ecosystems. *Nature Communications* 2016 7. 10.1038/ncomms10541 26817514 PMC4738359

[B19] Dias T. Dukes A. Antunes P. M. (2015). Accounting for soil biotic effects on soil health and crop productivity in the design of crop rotations. *J. Sci. Food Agric.* 95, 447–454. 10.1002/jsfa.6565 24408021

[B20] Edgar R. C. (2013). UPARSE: highly accurate OTU sequences from microbial amplicon reads. *Nat Methods* 10 996–998. 10.1038/nmeth.2604 23955772

[B21] Garat M. de Aurrecoechea I. Wisniewski M. Vero S. Garmendia G. (2010). Cystofilobasidium infirmominiatum as a biocontrol agent of postharvest diseases on apples and citrus. *International Symposium on Biological Control of Postharvest Diseases: Challenges and Opportunities* 905 169–180. 10.17660/ActaHortic.2011.905.18

[B22] Geyer K. M. Kyker-Snowman E. Grandy A. S. Frey S. D. (2016). Microbial carbon use efficiency: accounting for population, community, and ecosystem-scale controls over the fate of metabolized organic matter. *Biogeochemistry* 127 173–188. 10.1007/s10533-016-0191-y

[B23] Ghosh A. Bhattacharyya R. Dwivedi B. Meena M. Agarwal B. Mahapatra P. (2016). Temperature sensitivity of soil organic carbon decomposition as affected by long-term fertilization under a soybean based cropping system in a sub-tropical Alfisol. *Agriculture, Ecosystems & Environment* 233 202–213. 10.1016/j.agee.2016.09.010

[B24] Gu S. Xiong X. Tan L. Deng Y. Du X. Yang X. (2022). Soil microbial community assembly and stability are associated with potato (Solanum tuberosum L.) fitness under continuous cropping regime. *Frontiers in Plant Science* 13:1000045. 10.3389/fpls.2022.1000045 36262646 PMC9574259

[B25] Guo Y. Luo H. Wang L. Xu M. Wan Y. Chou M. (2021). Multifunctionality and microbial communities in agricultural soils regulate the dynamics of a soil-borne pathogen. *Plant Soil* 461 309–322. 10.1007/s11104-020-04826-4

[B26] Gustavsen G. W. (2021). Sustainability and potato consumption. *Potato Res.* 64, 571–586. 10.1007/s11540-021-09493-1

[B27] Han H. Hwang J. Kim G. (2021). Characterizing the origins of dissolved organic carbon in coastal seawater using stable carbon isotope and light absorption characteristics. *Biogeosciences* 18 1793–1801. 10.5194/bg-18-1793-2021

[B28] Han Z. Xu P. Li Z. Lin H. Zhu C. Wang J. (2022). Microbial diversity and the abundance of keystone species drive the response of soil multifunctionality to organic substitution and biochar amendment in a tea plantation. *GCB Bioenergy* 14 481–495. 10.1111/gcbb.12926

[B29] Hiltunen L. H. Tarvainen O. Kelloniemi J. Tanskanen J. Karhu J. Valkonen J. P. T. (2021). Soil bacterial community in potato tuberosphere following repeated applications of a common scab suppressive antagonist. *Applied Soil Ecology* 2021 167. 10.1016/j.apsoil.2021.104096

[B30] Hirsch A. M. Valdés M. (2010). Micromonospora: an important microbe for biomedicine and potentially for biocontrol and biofuels. *Soil Biol Biochem* 42 536–542. 10.1016/j.soilbio.2009.11.023

[B31] Hou Q. Wang W. Yang Y. Hu J. Bian C. Jin L. (2020). Rhizosphere microbial diversity and community dynamics during potato cultivation. *European Journal of Soil Biology* 98 103176. 10.1016/j.ejsobi.2020.103176

[B32] Jiang J. Song Z. Yang X. Mao Z. Nie X. Guo H. (2017). Microbial community analysis of apple rhizosphere around Bohai Gulf. *Sci Rep-Uk* 7 1–9. 10.1038/s41598-017-08398-9 28827532 PMC5566992

[B33] Karimi B. Dequiedt S. Terrat S. Jolivet C. Arrouays D. Wincker P. (2019). Biogeography of soil bacterial networks along a gradient of cropping intensity. *Sci Rep-Uk* 9 1–10. 10.1038/s41598-019-40422-y 30846759 PMC6405751

[B34] Kerfahi D. Tripathi B. M. Dong K. Go R. Adams J. M. (2016). Rainforest conversion to rubber plantation may not result in lower soil diversity of bacteria, fungi, and Nematodes. *Microbial Ecology* 72 359–371. 10.1007/s00248-016-0790-0 27221090

[B35] Lang M. Bei S. Li X. Kuyper T. W. Zhang J. (2019). Rhizoplane bacteria and plant species co-determine phosphorus-mediated microbial legacy effect. *Frontiers in microbiology* 10:2856. 10.3389/fmicb.2019.02856 31921037 PMC6914688

[B36] Larkin R. P. Halloran J. M. (2014). Management effects of disease-suppressive rotation crops on potato yield and soilborne disease and their economic implications in potato production. *Am J Potato Res* 91 429–439. 10.1007/s12230-014-9366-z

[B37] Li H. Li C. Song X. Liu Y. Gao Q. Zheng R. (2022). Impacts of continuous and rotational cropping practices on soil chemical properties and microbial communities during peanut cultivation. *Scientific reports* 12 1–12. 10.1038/s41598-022-06789-1 35177784 PMC8854431

[B38] Li J. Luo C. Zhang D. Song M. Cai X. Jiang L. (2018). Autochthonous bioaugmentation-modified bacterial diversity of phenanthrene degraders in PAH-contaminated wastewater as revealed by DNA-stable isotope probing. *Environ Sci Technol* 52 2934–2944. 10.1021/acs.est.7b05646 29378393

[B39] Li X. Jia K. He J. Li S. (2007). Isolation and identification of chlorpyrifos degrading strain Sphingomonas sp. Dsp-2 and its chlorpyrifos degradation characteristics. *Acta Pedologica Sinica* 44 734–739.

[B40] Li X. Lewis E. E. Liu Q. Li H. Bai C. Wang Y. (2016). Effects of long-term continuous cropping on soil nematode community and soil condition associated with replant problem in strawberry habitat. *Sci Rep* 6 30466. 10.1038/srep30466 27506379 PMC4978966

[B41] Lin C. Tsai C.-H. Chen P.-Y. Wu C.-Y. Chang Y.-L. Yang Y.-L. (2018). Biological control of potato common scab by Bacillus amyloliquefaciens Ba01. *PLoS One* 13:e0196520. 10.1371/journal.pone.0196520 29698535 PMC5919641

[B42] Ling N. Zhu C. Xue C. Chen H. Duan Y. Peng C. (2016). Insight into how organic amendments can shape the soil microbiome in long-term field experiments as revealed by network analysis. *Soil Biol Biochem* 99 137–149. 10.1016/j.soilbio.2016.05.005

[B43] Liu H. Pan F. Han X. Song F. Zhang Z. Yan J. (2019). Response of soil fungal community structure to long-term continuous soybean cropping. *Front Microbiol* 9:3316. 10.3389/fmicb.2018.03316 30687292 PMC6333693

[B44] Liu X. Li Y. Han B. Zhang Q. Zhou K. Zhang X. (2012). Yield response of continuous soybean to one-season crop disturbance in a previous continuous soybean field in Northeast China. *Field Crops Research* 138 52–56. 10.1016/j.fcr.2012.09.012

[B45] Liu X. Liu H. Ren D. Liu C. Zhang Y. Wang S. (2022). Interlinkages between soil properties and keystone taxa under different tillage practices on the North China Plain. *Applied Soil Ecology* 178 104551. 10.1016/j.apsoil.2022.104551

[B46] Liu Z. Liu J. Yu Z. Yao Q. Li Y. Liang A. (2020). Long-term continuous cropping of soybean is comparable to crop rotation in mediating microbial abundance, diversity and community composition. *Soil Till Res* 197 104503. 10.1016/j.still.2019.104503

[B47] Ma H. Zhou J. Ge J. Nie J. Zhao J. Xue Z. (2022). Intercropping improves soil ecosystem multifunctionality through enhanced available nutrients but depends on regional factors. *Plant and Soil* 2022 1–14. 10.1007/s11104-022-05554-7

[B48] Nong Q. Zhang W. Lan T. Su Q. Chen Y. Zhang Y. (2017). Screening and identification of dark septate Endophytestrain L-14 and its mechanism of banana fusarium Wilt disease resistance. *Chinese J Tropical Crops* 38 559–564.

[B49] Oberholster T. Vikram S. Cowan D. Valverde A. (2018). Key microbial taxa in the rhizosphere of sorghum and sunflower grown in crop rotation. *Science of the total environment* 624 530–539. 10.1016/j.scitotenv.2017.12.170 29268225

[B50] Ochieno D. M. Karoney E. M. Muge E. K. Nyaboga E. N. Baraza D. L. Shibairo S. I. (2021). Rhizobium-linked nutritional and phytochemical changes under multitrophic functional contexts in Sustainable Food Systems. *Front Sustain Food Syst* 4:604396. 10.3389/fsufs.2020.604396

[B51] Qi G. Zhu F. Du P. Yang X. Qiu D. Yu Z. (2010). Lipopeptide induces apoptosis in fungal cells by a mitochondria-dependent pathway. *Peptides* 31 1978–1986. 10.1016/j.peptides.2010.08.003 20713103

[B52] Qin S. H. Yeboah S. Cao L. Zhang J. L. Shi S. L. Liu Y. H. (2017). Breaking continuous potato cropping with legumes improves soil microbial communities, enzyme activities and tuber yield. *Plos One* 12:e0175934. 10.1371/journal.pone.0175934 28463981 PMC5413038

[B53] Rao D. Meng F. Yan X. Zhang M. Yao X. Kim K. S. (2021). Changes in soil microbial activity, bacterial community composition and function in a long-term continuous soybean cropping system after corn insertion and fertilization. *Front. Microbiol.* 12:638326. 10.3389/fmicb.2021.638326 33897643 PMC8059791

[B54] Ryan M. R. Crews T. E. Culman S. W. DeHaan L. R. Hayes R. C. Jungers J. M. (2018). Managing for multifunctionality in perennial grain crops. *Bioscience* 68 294–304. 10.1093/biosci/biy014 29662249 PMC5894082

[B55] Shi W. C. Li M. C. Wei G. S. Tian R. M. Li C. P. Wang B. (2019). The occurrence of potato common scab correlates with the community composition and function of the geocaulosphere soil microbiome. *Microbiome* 7:14. 10.1186/s40168-019-0629-2 30709420 PMC6359780

[B56] Sinsabaugh R. L. Lauber C. L. Weintraub M. N. Ahmed B. Allison S. D. Crenshaw C. (2008). Stoichiometry of soil enzyme activity at global scale. *Ecology letters* 11 1252–1264. 10.1111/j.1461-0248.2008.01245.x 18823393

[B57] Sugiyama A. Ueda Y. Zushi T. Takase H. Yazaki K. (2014). Changes in the bacterial community of soybean rhizospheres during growth in the field. *Plos One* 9:e100709. 10.1371/journal.pone.0100709 24955843 PMC4067361

[B58] Tan H. Li Q. Zhang H. Wu C. Zhao S. Deng X. (2020). Pesticide residues in agricultural topsoil from the Hainan tropical riverside basin: Determination, distribution, and relationships with planting patterns and surface water. *Science of the Total Environment* 722 137856. 10.1016/j.scitotenv.2020.137856 32208254

[B59] Tao C. Li R. Xiong W. Shen Z. Liu S. Wang B. (2020). Bio-organic fertilizers stimulate indigenous soil *Pseudomonas* populations to enhance plant disease suppression. *Microbiome* 8 137. 10.1186/s40168-020-00892-z 32962766 PMC7510105

[B60] Toju H. Tanabe A. S. Sato H. (2018). Network hubs in root-associated fungal metacommunities. *Microbiome* 6 1–16. 10.1186/s40168-018-0497-1 29935536 PMC6015470

[B61] Vick-Majors T. J. Priscu J. C. Amaral-Zettler L. (2014). Modular community structure suggests metabolic plasticity during the transition to polar night in ice-covered Antarctic lakes. *The ISME journal* 8 778–789. 10.1038/ismej.2013.190 24152712 PMC3960534

[B62] Wang B. Wang Y. Cui X. Zhang Y. Yu Z. (2019). Bioconversion of coal to methane by microbial communities from soil and from an opencast mine in the Xilingol grassland of northeast China. *Biotechnology for biofuels* 12 1–15. 10.1186/s13068-019-1572-y 31624498 PMC6781394

[B63] Wang Q. Garrity G. M. Tiedje J. M. Cole J. R. (2007). Naive Bayesian classifier for rapid assignment of rRNA sequences into the new bacterial taxonomy. *Appl Environ Microbiol* 73 5261–5267. 10.1128/AEM.00062-07 17586664 PMC1950982

[B64] Wang Q. Wang C. Yu W. Ali T. Chen D. Huang Y. (2018). Effects of nitrogen and phosphorus inputs on soil bacterial abundance, diversity, and community composition in Chinese fir plantations. *Frontiers in Microbiology* 9:1543. 10.3389/fmicb.2018.01543 30072961 PMC6060263

[B65] Wang X. Duan Y. Zhang J. Ciampitti I. A. Cui J. Qiu S. (2022). Response of potato yield, soil chemical and microbial properties to different rotation sequences of green manure-potato cropping in North China. *Soil Till Res* 217 105273. 10.1016/j.still.2021.105273

[B66] Wei W. Yang M. Liu Y. Huang H. Ye C. Zheng J. (2018). Fertilizer N application rate impacts plant-soil feedback in a sanqi production system. *Sci Total Environ* 633 796–807. 10.1016/j.scitotenv.2018.03.219 29602118

[B67] Wu X. Hu H. Wang R. Zhao J. Yang D. Wang L. (2021a). Effects of reduction of chemical fertilizer and substitution coupled with organic manureon the molecular ecological network of microbial communities in fluvo-aquic soil. *Acta Pedologica Sinica* 59 545–556.

[B68] Wu X. Zhang T. Zhao J. Wang L. Yang D. Li G. (2021b). Variation of soil bacterial and fungal communities from fluvo-aquic soil under chemical fertilizer reduction combined with organic materials in North China Plain. *Journal of Soil Science and Plant Nutrition* 21 349–363. 10.1007/s42729-020-00365-0

[B69] Wunsch M. J. Bergstrom G. C. (2011). Genetic and morphological evidence that Phoma sclerotioides, causal agent of brown root rot of alfalfa, is composed of a species complex. *Phytopathology* 101 594–610. 10.1094/PHYTO-04-10-0107 20955081

[B70] Xu J. Xu X. Wang L. Jiang Y. Zhang W. Cao Y. (2014). Biological characteristics of tomato wilt fungus. *Journal of Shenyang Aricultural University* 6 673–678.

[B71] Xu X. Zhang L. Yang X. Cao H. Li J. Cao P. (2022). Alternaria spp. associated with leaf blight of maize in Heilongjiang Province. *China. Plant Dis* 106 572–584. 10.1094/PDIS-06-21-1151-RE 34472972

[B72] Yan L. Zhang W. Duan W. Zhang Y. Zheng W. Lai X. (2021). Temporal bacterial community diversity in the nicotiana tabacum rhizosphere over years of continuous monocropping. *Front Microbiol* 12:1276. 10.3389/fmicb.2021.641643 34113322 PMC8186668

[B73] Yan Z. Hao Z. Wu H. Jiang H. Yang M. Wang C. (2019). Co-occurrence patterns of the microbial community in polycyclic aromatic hydrocarbon-contaminated riverine sediments. *Journal of hazardous materials* 367 99–108. 10.1016/j.jhazmat.2018.12.071 30594728

[B74] Yang T. Evans B. Bainard L. D. (2021). Pulse frequency in crop rotations alters soil microbial community networks and the relative abundance of fungal plant pathogens. *Frontiers in microbiology* 12:667394. 10.3389/fmicb.2021.667394 34122380 PMC8189174

[B75] Yao Z. Zhang J. Du Y. Liu Y. Zhang L. (2020). Productivity evaluation of crop rotation in cold and arid region of Northern China. *Acta Agronomica Sinica* 16 32–34.

[B76] Zhang K. Wang X. Sun J. Yang Q. Sun H. (2017). Identification of potato rot disease inner ventilation bank of Huade County, Mongilia during storage period. *J Anhui Sci* 45 147–151.

[B77] Zhao L. Liu X. Zhang B. Zhang Y. Li C. Qi J. (2013). “A biocontrol strain isolation, identification and antimicrobial activity,” in *Proceedings of the Chinese Society for Plant Protection.*

[B78] Zhao W. Guo Q. Su Z. Wang P. Dong L. Hu Q. (2021). Characterization of fungal community structure in the rhizosphere soil of healthy and diseased-Verticillium Wilt potato plants and carbon source utilization. *Scientia Agricultura Sinica* 54 296–309.

[B79] Zheng Q. Hu Y. Zhang S. Noll L. Böckle T. Dietrich M. (2019). Soil multifunctionality is affected by the soil environment and by microbial community composition and diversity. *Soil Biology and Biochemistry* 136 107521. 10.1016/j.soilbio.2019.107521 31700196 PMC6837881

[B80] Zhou H. Peng Y. Li T. Xie Y. Tang L. Wang R. (2019). Effects of potato continuous cropping on soil physicochemical and biological properties. *J. Hunan Agric. Univ.* 45 611–616.

[B81] Zhou X. Wu F. (2018). Vanillic acid changed cucumber (Cucumis sativus L.) seedling rhizosphere total bacterial, *Pseudomonas* and Bacillus spp. communities. *Sci Rep* 8 1–11. 10.1038/s41598-018-23406-2 29563548 PMC5862977

