# Supplementary material for: Crop rotations increased soil ecosystem multifunctionality by improving keystone taxa and soil properties in potatoes
Source: Front Microbiol. 2023 Feb 23;14:1034761. doi: 10.3389/fmicb.2023.1034761 (PMC9995906; doi:10.3389/fmicb.2023.1034761)
Supplement: Supplementary file 1 [file Data_Sheet_1.doc]

Supplementary Material

**Supplementary Figures and Tables**

**Table S1** Illumina MiSeq sequenced fungal data (at 97% sequence similarity) based on the 16S and ITS rRNA gene.

**Table S2** The co-occurrence network topological properties of soil bacterial and fungal communities.

**Table S3** The percent of different categories within *Zi*-*Pi* plots.

**Table S4** Genera display difference in *Zi*-*Pi* plots among different cropping systems.

**Table S5** Pearson correlation coefficients between the soil properties and the abundances of the dominant and keystone taxa.

**Table S6** Pearson correlation coefficients between the soil biotic and abiotic factors and the single soil functions and multifunctionality.

**Table S7.** Results of structural equation modeling of cropping system effects on soil ecosystem multifunctionality through all plausible interaction pathways.

**Table S8** Pearson correlation coefficients between the keystone taxa abundance and the pathogenic microbe abundance.

**Fig. S1.** Indicator bacteria with LDA scores of 2.5 or greater (A) and fungi with LDA scores of 4.0 or greater (B) associated with soil from different crop rotations.

**Table S1** Illumina MiSeq sequenced fungal data (at 97% sequence similarity) based on the 16S and ITS rRNA gene.

| Amplified Region | Sequences | Bases(bp) | Average Length | Min length | Max length |
| --- | --- | --- | --- | --- | --- |
| 338F_806R | 980851 | 408589145 | 417 | 259 | 496 |
| ITS1F_ITS2R | 754215 | 178568114 | 237 | 148 | 494 |

**Table S2** The co - occurrence network topological properties of soil bacterial and fungal communities.

| Microbe | Treatments | Nodes | Edges | Network density | Modularity | Average clustering coefficient | Average path length |
| --- | --- | --- | --- | --- | --- | --- | --- |
| Bacteria | PP | 195 | 1858 | 0.098 | 1.033 | 0.632 | 3.486 |
|  | PO | 193 | 2254 | 0.122 | 7.24 | 0.636 | 3.383 |
|  | PFM | 195 | 2984 | 0.158 | 2.46 | 0.669 | 3.284 |
| Fungi | PP | 110 | 561 | 0.094 | 5.781 | 0.612 | 3.703 |
|  | PO | 95 | 427 | 0.096 | 4.384 | 0.597 | 3.75 |
|  | PFM | 113 | 685 | 0.108 | 5.691 | 0.609 | 3.537 |

Note: PP, potato continuous cropping; PO, potato - oat rotation; PFM, potato - forage maize rotation.

**Table S3** The percent (%) of different categories within *Zi*-*Pi* plots.

| Microbe | Treatments | Peripherals | Connectors | Module hubs | Network hubs |
| --- | --- | --- | --- | --- | --- |
| Bacteria | PP | 24.23 | 74.74 | 1.03 | 0.00 |
|  | PO | 4.35 | 95.65 | 0.00 | 0.00 |
|  | PFM | 10.31 | 89.69 | 0.00 | 0.00 |
| Fungi | PP | 6.42 | 93.58 | 0.00 | 0.00 |
|  | PO | 4.84 | 95.16 | 0.00 | 0.00 |
|  | PFM | 3.57 | 95.24 | 0.00 | 1.19 |

Note: PP, potato continuous cropping; PO, potato - oat rotation; PFM, potato - forage maize rotation.

**Table S4 Keystone genera display difference in *Zi*-*Pi* plots among different cropping systems.**

| Genera | Within module connectivities | | | Among module connectivities | | | Degree | | | Modularity | | |
| --- | --- | --- | --- | --- | --- | --- | --- | --- | --- | --- | --- | --- |
|  | PP | PO | PFM | PP | PO | PFM | PP | PO | PFM | PP | PO | PFM |
| *Nitrospira.1* | 1.48 | 0.88 | -0.27 | 0.43 | 0.87 | 0.96 | 16 | 41 | 11 | 3 | 5 | 2 |
| *Lysinibacillus* | 1.79 | -0.13 | 0.19 | 0.48 | 0.73 | 0.69 | 21 | 18 | 28 | 11 | 6 | 8 |
| *Microlunatus.1* | -0.51 | 0.42 | 0.77 | 0.59 | 0.63 | 0.75 | 7 | 18 | 63 | 10 | 5 | 5 |
| *Sphingomonas.3* | 1.35 | -0.60 | 1.15 | 0.46 | 0.87 | 0.77 | 29 | 18 | 53 | 4 | 6 | 5 |
| *Bryobacter.1* | 0.50 | 2.20 | NA | 0.59 | 0.75 | 0.89 | 25 | 33 | 9 | 2 | 4 | 0 |
| *Micromonospora* | -0.99 | NA | 0.26 | 0.41 | 0.92 | 0.71 | 21 | 25 | 25 | 10 | 0 | 5 |
| *Schizothecium.1* | 0.00 | NA | 0.17 | 0.44 | 0.95 | 0.91 | 8 | 10 | 11 | 6 | 0 | 3 |
| *Cystofilobasidium* | NA | NA | 3.41 | NA | NA | 0.66 | NA | NA | 18 | NA | NA | 4 |

Note: PP, potato continuous cropping; PO, potato - oat rotation; PFM, potato - forage maize rotation.

**Table S5** Pearson correlation coefficients between the soil properties and the abundances of the dominant and keystone taxa.

|  |  | C/N | pH | TN | TP | AP |
| --- | --- | --- | --- | --- | --- | --- |
| Dominant bacteria | *Cyanobacteria* | -0.516* | -0.473 | -0.386 | 0.596* | 0.595* |
|  | *Bacilli* | 0.541* | 0.128 | 0.072 | 0.033 | 0.052 |
|  | *Gemmatimonadetes* | 0.130 | 0.491 | 0.662** | -0.462 | -0.381 |
| Dominant fungi | *Nectriaceae* | -0.193 | -0.636* | -0.547* | 0.754** | 0.804** |
|  | *Chaetomium* | 0.066 | 0.596* | 0.527* | -0.787** | -0.763** |
|  | *Basidiomycota* | 0.500 | 0.760** | 0.534* | -0.707** | -0.783** |
| Keystone taxa | *Nitrospira1* | -0.022 | 0.359 | 0.549* | -0.153 | -0.105 |
|  | *Lysinibacillus* | 0.371 | 0.289 | 0.485 | -0.241 | -0.138 |
|  | *Sphingomonas3* | 0.257 | 0.320 | 0.614* | -0.320 | -0.100 |
|  | *Bryobacter1* | 0.638* | 0.127 | 0.324 | -0.008 | 0.158 |
|  | *Schizothecium1* | 0.481 | 0.781** | 0.514* | -0.597* | -0.536* |
|  | *Cystofilobasidium* | 0.691** | 0.604* | 0.367 | -0.351 | -0.463 |

Note: **P* < 0.05, ***P* < 0.01, and ****P* < 0.001

**Table S6** Pearson correlation coefficients between the soil biotic and abiotic factors and the single soil functions and multifunctionality.

|  | N cycle | P cycle | C cycle | EMF |
| --- | --- | --- | --- | --- |
| pH | 0.715** | -0.618* | 0.755** | 0.641* |
| TN | 0.727** | -0.592* | 0.604* | 0.544* |
| AN | 0.514 | -0.094 | 0.382 | 0.621* |
| NAG | 0.842** | -0.792** | 0.846** | 0.663** |
| TP | -0.827** | 0.883** | -0.850** | -0.578* |
| AP | -0.718** | 0.887** | -0.803** | -0.454 |
| SOM | 0.709** | -0.599* | 0.782** | 0.675** |
| GC | 0.668** | -0.688** | 0.780** | 0.567* |
| *Schizothecium*1 | 0.598* | -0.468 | 0.580* | 0.534* |
| *Pyrenochaetopsis* | 0.241 | -0.533* | 0.397 | 0.060 |
| *Chaetomium* | 0.705** | -0.817** | 0.866** | 0.557* |
| *Verticillium* | -0.390 | 0.450 | -0.527* | -0.350 |
| *Alternaria* | -0.571* | 0.682** | -0.764** | -0.487 |
| *Fusarium* | -0.610* | 0.762** | -0.661** | -0.362 |
| *Gibberella* | -0.661** | 0.712** | -0.735** | -0.503 |
| *Plectosphaerella* | -0.741** | 0.734** | -0.830** | -0.624* |
| *Phoma* | -0.700** | 0.850** | -0.780** | -0.453 |
| *Lectera* | -0.813** | 0.825** | -0.845** | -0.613* |
| *Colletotrichum* | -0.621* | 0.728** | -0.725** | -0.451 |
| N cycle | 1.000 | -0.715** | 0.880** | 0.874** |
| P cycle | -0.715** | 1.000 | -0.822** | -0.372 |
| C cycle | 0.880** | -0.822** | 1.000 | 0.795** |

Note: **P* < 0.05, ***P* < 0.01

**Table S7.** Results of structural equation modeling of cropping system effects on soil ecosystem multifunctionality through all plausible interaction pathways. The table shows the unstandardized path coefficients (estimates), standard error of regression weight (S.E.), the critical value for the regression weight (C.R.), and level of significance for the regression weight (*P*). *** indicates *P* ≤ 0.001, ** indicates *P* ≤ 0.01.

|  | Path | |  | | Estimate | S.E. | C.R. | *P* |
| --- | --- | --- | --- | --- | --- | --- | --- | --- |
| pH | | <--- | | Cropping system | 0.788 | 0.163 | 4.832 | *** |
| SOC | | <--- | | Cropping system | 0.580 | 0.216 | 2.689 | ** |
| Pathogenic microbes | | <--- | | Cropping system | -0.852 | 0.139 | -6.144 | *** |
| Keystone taxa | | <--- | | pH | 0.781 | 0.166 | 4.717 | *** |
| EMF | | <--- | | SOC | 0.656 | 0.089 | 7.401 | *** |
| EMF | | <--- | | Pathogenic microbes | -0.164 | 0.105 | -1.562 | 0.118 |
| EMF | | <--- | | Keystone taxa | 0.489 | 0.087 | 5.644 | *** |

Note: EMF, soil ecosystem multifunctionality.

**Table S8** Pearson correlation coefficients between the keystone taxa abundance and the pathogenic microbe abundance.

|  | *Schizothecium1* | *Cystofilobasidium* |
| --- | --- | --- |
| *Alternaria* | -.603* | -0.119 |
| *Gibberella* | -.555* | -0.397 |
| *Plectosphaerella* | -.594* | -0.433 |
| *Phoma* | -.578* | -0.41 |
| *Lectera* | -.676** | -0.45 |
| *Streptomyces scabiei* | -0.207 | -.645** |

Note: **P* < 0.05, ***P* < 0.01

**Fig. S1.** Indicator bacteria with LDA scores of 2.5 or greater (A) and fungi with LDA scores of 4.0 or greater (B) associated with soil from different crop rotations. PP, potato continuous cropping; PO, potato - oat rotation; PFM, potato - forage maize rotation.
